# The link between problematic internet use, problematic gaming, and psychological distress: does sleep quality matter?

**DOI:** 10.1186/s12888-021-03105-5

**Published:** 2021-02-16

**Authors:** Qian Wang, Komi Mati, Yong Cai

**Affiliations:** 1 School of Public Health, Shanghai Jiao Tong University School of Medicine, 227 S Chongqing Rd, Huangpu District, Shanghai, 200025 China; 2 Econometrica, Inc., 7475 Wisconsin Avenue, Suite 1000, Bethesda, MD 20814 USA

**Keywords:** Problematic internet use, Problematic gaming, Sleep quality, Psychological distress, PROCESS macro, Mediation

## Abstract

**Introduction:**

This study aimed to examine the mediating role of sleep quality in the association of problematic internet use (PIU) and problematic gaming with psychological distress among college students in China.

**Methods:**

Data of 1040 full-time students from multiple colleges in China were examined. Respondents were asked about their internet use and gaming behaviors, sleep quality, psychological distress, and sociodemographic characteristics. The mediating role of sleep quality in the PIU- and problematic gaming-psychological distress link was examined respectively.

**Results:**

PIU was associated with decreased sleep quality (*r* = .32, *p* < .001) and increased psychological distress (*r* = .46, *p* < .001). Problematic gaming was also associated with decreased sleep quality (*r* = .22, *p* < .001) and increased psychological distress (*r* = .46, *p* < .001). Sleep quality accounted for 23.5% of the indirect effect of PIU on psychological distress, and 17.9% of the indirect effect of problematic gaming on psychological distress.

**Conclusions:**

Sleep quality had a meaningful mediating effect on the PIU-psychological distress link, but only exerted a small mediating effect on the problematic gaming-psychological distress link. In addition to promoting healthy internet usage, strategies aimed at mitigating the negative effect of excessive internet use on psychological health might benefit from those aimed at improving sleep quality.

## Introduction

By June of 2019, Asia had approximately 2.3 billion internet users, making up half (50.7%) of all internet users worldwide [1]. Within Asia, China was the country with the highest percentage of internet users (37.1%) among its population [2]. By March of 2020, there were 904 million internet users in China, and the largest percentage of users (21.5%) were those between 20 and 29 years of age, or the young adult population [3]. The current internet coverage rate was 64.5% in China [3], still lower than the rates in countries such as the United States and Japan [2]. It is expected that, with increased internet coverage, the number of internet users in China would likely rise. As internet use becomes more prevalent, the likelihood of excessive use has also increased.

Griffiths and other scholars have recognized the addictive potential of technology use as early as the 1990s, and in a series of papers, they discussed whether excessive internet use can be deemed as an “addiction” [4, 5]. Yet, the literature on excessive internet use has evolved considerably since, more recent view about excessive internet use seems to be that, rather than a stand-alone addiction, excessive internet use, as problematic internet use (PIU), is more of an umbrella term depicting a range of behaviors conducted via the internet, such as video gaming, shopping, social networking, pornography viewing, cyber-bullying, etc. [6]. Thus, it might be helpful to differentiate “generalized internet addiction” from “specific internet addiction” [7].

Consistent with this view, Gaming Disorder (GD), either online or offline, is now included in the 11th revision of the International Classification of Diseases (ICD-11) developed by the World Health Organization (WHO) [8]. It depicts a pattern of gaming behavior (i.e., digital or video gaming) featuring impaired control over gaming, increased priorities given to gaming over other interests or activities, continuation or escalation of gaming in spite of negative consequences [8]. To be diagnosed, the behavior has to be severe enough to cause significant impairments in personal, social, occupational, or other functional domains lasting for 12 months [9]. Earlier, the American Psychological Association (APA) proposed that Internet Gaming Disorder (IGD), a sub-type of PIU, should be included under Conditions Warranting More Experience and Research in the 5th edition of the Diagnostic and Statistical Manual of Mental Disorders (DSM-V) [10]. Some have argued that the conceptualization of IGD under the DSM-V might lack clarity as IGD was considered to include both internet gaming as well as non-Internet computerized gaming despite having the word “Internet” in its nomenclature [7].

Despite the debate on conceptual differences, two meta-analytic studies reported the pooled prevalence of PIU in the college student population in China to be approximately 11% [11, 12], higher than the average rates reported in western countries [13] Meanwhile, playing video or computer games is a common and popular leisure activity among college students worldwide including in China. The latest statistics indicated that, online game companies had the largest share of the market among all listed companies in China, while 532 million or over half (58.9%) of internet users in China were also internet game users [3]. For some students, gaming is as much part of their everyday life as studying and eating. Compared with the amount of research on PIU, there are fewer studies examining problematic gaming among young adults in mainland China. Several studies examining problematic gaming among university students in Macao and Hong Kong reported varied prevalence, ranging from 14.8% among respondents in Macao to 25.7% among respondents in Hong Kong [14, 15]. The prevalence of problematic gaming also varied from 2.1% among college students in Beijing to 7.41% among college students in Luzhou, Sichuan [16, 17]. The varied prevalence rate could be attributed to differences in methodologies or instruments utilized in measuring problematic gaming, or a lack of well-designed studies testing measurement invariance of the same instrument [18].

Both PIU and problematic gaming constitute a growing public health concern among the college student population in China. Compared to high school students, college students are relieved of the tremendous pressure from preparing for and sitting through the Chinese college entrance exam or Gaokao, they typically devote more time to pursuing other non-academic activities. A study comparing health behaviors of adolescents and college students in China found college students reported more screen-time on average than both middle school and high school students [19]. Another study comparing prevalence of problematic gaming among adolescents and college students found a higher prevalence among college students [16]. Spending excessive time playing games can be detrimental. Both PIU and problematic gaming have been linked to symptoms of psychopathology. A meta-analytic study found PIU linked to depression, anxiety, attention deficit/hyperactivity disorder (ADHD), obsessive-compulsive symptoms (OCS), and/or hostility/aggression [20]. Several studies including meta-analytic reviews found problematic gaming associated with similar psychopathologies such as depression, obsessive-compulsive symptoms, ADHD, and anxiety [21, 22].

Sleep not only plays a critical role in maintaining our physical and mental health [23], but it is also increasingly recognized for its contribution to various psychopathologies [20]. More specially, problems with sleep can act as an outcome as well as a cause of mental disorders [24]. Sleep problems often occur as a result of PIU, it could enhance addictions to internet usage, and mediate further psychiatric morbidities [25]. Sleep quality typically reflects the overall experience of sleep, while sleep duration or quantity reflects the amount of time an individual spends sleeping. Some evidence seems to suggest that sleep duration such as night time sleep might be a poor indicator for screening PIU, as individuals might compensate for reduced night time sleep duration by taking naps during the day [26]. In the seven studies (Table 1) that examined mediation models with different orderings of PIU, problematic gaming, sleep problems, and psychological symptoms, majority of the studies assessed the mediating role of sleep quality in the PIU-psychological health link or the problematic gaming-psychological health link [27–29, 32, 33]. For example, Bhandari et al. found sleep quality seemed to be a stronger mediator, mediating 30.9% of the indirect effect of PIU on depressive symptoms, whereas PIU was a weaker mediator, mediating only 16.5% of the indirect effect of sleep quality on depressive symptoms [28]. Only two studies examined sleep problems as a mediator of the problematic gaming-psychological health relation [27, 33], and the effect size varied considerably. A review study found insufficient evidence supporting a consistent sleep pathway between problematic gaming and depression [34]. The lack of supporting evidence has been attributed to the recognition of addictive gaming as a disorder only recently, and a lack of studies using validated instruments to assess problematic gaming as well as sleep quality [34]. These findings suggested that, the mediating role of sleep quality in the PIU/problematic gaming and psychological health connection warrants further study.

**Table 1:** Studies assessing the mediating role of sleep, problematic internet use (PIU), problematic gaming, and psychological symptoms

First author, year	Country	Participants	Predictor variable	Mediator	Outcome variable
Li, 2019 [27]	United States	Adolescents	Social messaging Web surfingTV/movie watching Gaming	Problems falling alseep;Problems staying asleep;Weekday sleep duration	Depressive symptoms
Bhandari, 2017 [28]	Nepal	Undergraduatestudents	Sleep quality	Internet addiction	Depressive symptoms
			Internet addiction	Sleep quality	Depressive symptoms
An, 2014 [29]	China	Adolescents	PIU	Sleep quality	Psychological symptoms
Tan, 2016 [30]	China	Adolescents	PIU	Depressive symptoms	Sleep disturbance
			Depressive symptoms	PIU	Sleep disturbance
Guo, 2018 [31]	China	Adolescents	PIU	Sleep disturbance	Suicidal behavior
			Suicidal behavior	Sleep disturbance	PIU
Lemola, 2015 [32]	Switzerland	Adolescents	Electronic media use before bed	Sleep duration Sleep difficulties	Depressive symptoms
Guerrero, 2019 [33]	United States	Children	Screen time (i.e. playing video games)	Sleep duration	Emotional/behavioralproblems

Therefore, this study aimed to address this issue by examining the mediating role of sleep quality in the PIU-psychological distress link and the problematic gaming-psychological distress link using widely-used instruments. College students in China were the population under study mainly for two reasons: first, they belong to the age group that reports greater internet usage and problems associated with it [35]; second, they tend to experience varied levels of sleep problems, with the prevalence rate ranging between 14.3 and 42.6% [36, 37]. By examining the phenomenon in one of China’s internet-savvy populations, this study may contribute to further understanding of the complex relationship between PIU, problematic gaming, sleep quality, and overall psychological distress, and to aid in the identification of pathways for intervention.

## Materials and methods

### Participants

A total of 1071 students from multiple colleges in China were recruited initially through word-of-mouth and friend referral, then through snowball sampling. The study was approved by the Ethics Committee at an urban medical university in the Northeast of China. All participants were informed of the purpose of the study and signed written informed consent before completing an online survey. More details about the study were described in a previous article [38]. Only data from full-time students between 16 and 26 years of age who completed the survey in full were included in final analysis. The final sample consisted of 1040 students, with 416 males (40.0%) and 624 females (60.0%) ranging in age between 16 and 26 (mean = 20.32; SD = 1.43). The sample had a maximum estimated sampling error of ±3.04% with a 95% confidence probability [39].

### Measures

#### Overall psychological distress

Overall psychological distress was assessed by the Symptom Checklist-90-Revised (SCL-90-R) [40]. The SCL-90-R is a widely used self-report questionnaire consisting of 90 items that attempt to gauge nine dimensions of psychiatric symptoms: somatization, obsessive compulsiveness, interpersonal sensitivity, depression, anxiety, hostility, phobic anxiety, paranoid ideation, and psychoticism. Each item is assessed on a 5-point Likert scale (0 = not at all, 4 = extremely), and the total score ranged between 0 and 360, with higher scores indicating higher level of overall psychological distress. The Chinese version of the scale has been shown to have good reliability and validity in the adolescent and young adult population [41]. In the current study, the McDonald’s omega reliability coefficient of the scale was 0.98 [42].

#### Problematic internet use (PIU)

PIU was assessed by the Internet Addiction Test (IAT) [43]. The IAT consists of 20 self-report items, each scored on a 5-point Likert scale (1 = never, 5 = always), yielding a total score between 20 and 100, with higher scores reflecting greater severity of PIU. The Chinese version of the IAT has been consistently found to have adequate reliability and validity among adolescents [44]. The McDonald’s omega reliability coefficient of the scale was 0.94 [42].

#### Problematic gaming

Problematic gaming was assessed by the 7-item Gaming Addiction Scale (GAS) [45]. The scale was a shorter version of the original 21-item scale developed based on the 7 criteria for diagnosing pathological gambling under the DSM-IV-R [45]. Each of the 7 items is rated on a 5-point Likert scale (1 = never, 5 = very often), yielding a total score between 7 and 35, with higher scores representing higher level of problematic gaming. Chinese version of the scale has been found to have good reliability and validity among adolescents [46, 47]. In the current study, the McDonald’s omega reliability coefficient of the scale was 0.95 [42].

#### Sleep quality

Sleep quality was assessed by the Pittsburg Sleep Quality Index (PSQI). The PSQI is a self-report measure consisting of 18 items measuring 7 dimensions of sleep: subjective sleep quality, sleep latency, sleep duration, habitual sleep efficiency, sleep disturbances, use of sleeping medication, and daytime dysfunction. The total score of the 7 dimensions ranges between 0 and 21, with higher scores indicating decreased sleep quality. The Cronbach’s alpha coefficient of the scale was 0.64 in the current study, consistent with values reported in previous studies [48], and the value of McDonald’s omega reliability coefficient of the scale was 0.67 [42]. Composite reliability (a less biased estimate of reliability) of the scale was 0.78, greater than the recommended minimum value of 0.7 [49].

#### Covariates

Covariates included gender (1 = male, 2 = female), class standing (1 = year one, 5 = year five), academic achievement (1 = top 20%, 2 = average, 3 = bottom 20%, 4 = unknown), annual household income (1 = < 50,000 yuan, 2 = 50,000–100,000 yuan, 3 = 100,001–200,000 yuan, 4 = 200,001–500,000 yuan, 5 = > 500,001 yuan), father’s education (1 = primary school or below, 2 = secondary school, 3 = high school/equivalent, 4 = junior/vocational college, 5 = 4-year college or above), mother’s education (1 = primary school or below, 2 = secondary school, 3 = high school or equivalent, 4 = junior/vocational college, 5 = 4-year college or above), number of days engaging in moderate to vigorous physical activity for at least 60 min per day within the past week (0 to 7 days), and substance use within the past 12 months (none, 1 type, 2 or more types).

### Data analysis

Values of skewness and kurtosis were acceptable (skewness < 2 and kurtosis < 4) for measures of PIU, problematic gaming, sleep quality, and psychological distress [50], indicating the distribution of the sample was nearly normal (Table 2). Frequencies were calculated for categorical variables, mean and standard errors were calculated for continuous variables. Bivariate correlation coefficients were calculated for PIU, problematic gaming, sleep quality, and psychological distress. Univariate analysis was performed exploring the bivariate association between each covariate and PIU, problematic gaming, sleep quality and psychological distress respectively. Next, Baron and Kenny’s method of testing mediation was used to assess whether the association between PIU or problematic gaming and psychological distress was mediated by sleep quality. According to Baron and Kenny, four conditions are required to establish a mediation effect [51]. First, PIU and problematic gaming (independent variable) must be significantly correlated with psychological distress (i.e., dependent variable; path c). Second, PIU and problematic gaming must be significantly correlated with sleep quality (i.e., mediator; path a). Third, sleep quality must be significantly correlated with psychological distress (path b). Fourth, mediation occurs when significance of the association between PIU or problematic gaming and psychological distress (path c) is weakened or becomes non-significant. A full mediation occurs when the association between PIU or problematic gaming and psychological distress (path c) is reduced to zero. Mediation analysis was performed using the PROCESS macro for SPSS developed by Hayes [52]. To test the significance of the indirect effect, a 10,000-sample bootstrapping procedure was used to estimate bias-corrected 95% confidence intervals (CIs). To determine the significance of the mediation effect, the following two criteria were used: first, mediation effect was deemed significant when the confidence intervals did not cross zero [52]; second, as a rule of thumb, partial mediation occurred when the ratio of indirect-to-total effect, also known as variance accounted for (VAF) value, was between 20 and 80%, and no mediation occurred when the VAF value was below 20% [53]. For each mediation analysis, three models were tested: the unadjusted model (model I), the model adjusted for sociodemographic (gender, class standing, academics, annual household income, father’s education, mother’s education), and the model adjusted for both sociodemographic and behavioral covariates (gender, class standing, academics, annual household income, father’s education, mother’s education, plus level of physical activity and substance use). All analyses were performed using IBM SPSS version 21.0.

**Table 2:** Sample characteristics (*N =* 1040)

Variable	N/Mean	%(N)/SD
Gender		
Male	416	40.0%
Female	624	60.0%
Class standing		
1st year	264	25.4%
2nd year	491	47.2%
3rd year and above	285	27.4%
Annual family income		
< 50,000 yuan	241	23.2%
50,000 to < 100,000 yuan	309	29.7%
100,000 to < 200,000 yuan	302	29.0%
> 200,000 yuan	188	18.1%
Academic achievement		
Top 20%	323	31.1%
Average	564	54.2%
Bottom 20%	153	14.7%
Father’s education		
Junior high school or below	381	36.6%
Senior high school	258	24.8%
College and above	401	38.6%
Mother’s education		
Junior high school or below	436	41.9%
Senior high school	250	24.0%
College and above	354	34.0%
Physical activity	2.36	0.06
0 days/week	210	20.2%
1 days/week	189	18.2%
2 days/week	212	20.4%
3 days/week	200	19.2%
4–7 days/week	229	22.0%
Substance use		
None	736	70.8%
1 type	137	13.2%
2 or more types	167	16.1%
Problematic internet use	54.09	0.51
*(Skewness, kurtosis)*	*(0.39)*	*(0.05)*
Problematic gaming	16.41	0.22
*(Skewness, kurtosis)*	*(0.87)*	*(−0.38)*
Sleep quality	5.45	0.09
*(Skewness, kurtosis)*	*(0.52)*	*(0.11)*
Psychological distress	54.65	2.05
*(Skewness, kurtosis)*	*(1.69)*	*(2.91)*
Subscales		
Somatization	6.07	0.27
Obsessive compulsiveness	8.09	0.25
Interpersonal sensitivity	6.38	0.23
Depression	8.81	0.33
Anxiety	5.58	0.24
Hostility	3.40	0.14
Phobic anxiety	3.35	0.16
Paranoid ideation	3.31	0.14
Psychoticism	5.62	0.24

## Results

### Sample characteristics

Sample characteristics are presented in Table 2. Majority of the sample were in their first and second year of college (72.6%), and were average or above-average in academic performance (85.3%). Approximately half (47.1%) of the sample came from families with an annual household income of 100,000 yuan ($14,286 USD equivalent) or above, and 38. 6% of the sample had a father with college-level education or beyond. Majority of the sample did not use any substances including alcohol and tobacco in the past year, yet, 16.1% reported using at 2 or more types of substances in the past year. Only less than a quarter (22.0%) of the sample were physically active for at least 4 days within the past week.

Except for gender and class standing, the mean score of PIU differed significantly across all other covariates (Table 3). The mean score of PIU was highest among those with low annual family income (< 50,000 yuan), poor academic achievement (bottom 20%), low maternal education level (junior high school or below), no physical activity during the past 7 days, and using 2 or more types of substances (Table 3). The mean score of problematic gaming was highest among those who were male, in third year or above, bottom 20% of their class, had low maternal educational level (junior high school or below), and used 2 or more types of substances in the past year (Table 3). The mean score of sleep quality was higher among students in their third year or above, had low annual family income, were physically active for 2 days or less per week, or used 2 or more types of substances in the past year (Table 3). And the mean score of psychological distress was higher among those in their third year or above, had low annual family income (< 50,000 yuan) and low parental education levels (junior high school or below), or used 2 or more types of substances in the past year (Table 3).

**Table 3:** Univariate association of problematic internet use (PIU), problematic gaming, sleep quality, and psychological distress with sample characteristics (*N =* 1040)

Variable	N	Problematic internet use		Problematic gaming		Sleep quality		Psychological distress	
		Mean	*p* value	Mean	*p* value	Mean	*p* value	Mean	*p* value
Gender									
Male	416	53.67	0.509	18.59	< 0.001	5.42	0.758	59.16	0.082
Female	624	54.37		14.95		5.47		51.64	
Class standing									
1st year	264	53.96	0.157	16.30	0.014	5.02	0.010	53.42	0.005
2nd year	491	53.28		15.89		5.50		49.26	
3rd year and above	285	55.61		17.42		5.76		65.06	
Annual family income									
< 50,000 yuan	241	56.71	0.031	16.91	0.419	6.05	0.003	69.75	< 0.001
50,000 to < 100,000 yuan	309	53.82		16.49		5.25		60.07	
100,000 to < 200,000 yuan	302	52.70		15.90		5.18		41.67	
> 200,000 yuan	188	53.41		16.46		5.45		47.22	
Academic achievement									
Top 20%	323	53.76	0.046	16.11	0.034	5.36	0.536	54.77	0.103
Average	564	53.47		16.21		5.44		51.85	
Bottom 20%	153	57.09		17.78		5.68		64.68	
Father’s education									
Junior high school or below	381	54.57	0.014	16.97	0.002	5.70	0.056	65.10	< 0.001
Senior high school	258	56.05		17.07		5.47		55.32	
College and above	401	52.38		15.45		5.20		44.28	
Mother’s education									
Junior high school or below	436	55.30	0.005	17.06	0.001	5.68	0.097	62.07	0.001
Senior high school	250	55.24		16.82		5.34		55.33	
College and above	354	51.79		15.32		5.25		45.02	
Physical activity									
0 days/week	210	57.33	< 0.001	15.95	0.133	5.60	0.027	55.67	0.502
1 days/week	189	55.06		16.59		5.80		56.68	
2 days/week	212	53.78		16.91		5.68		58.99	
3 days/week	200	55.26		17.12		5.21		54.47	
4–7 days/week	229	49.59		15.60		5.02		48.17	
Substance use									
None	736	52.57	< 0.001	14.91	< 0.001	5.15	< 0.001	39.42	< 0.001
1 type	137	52.78		16.99		5.65		46.74	
2 or more types	167	61.86		22.54		6.63		128.25	

PIU was associated with decreased sleep quality (*r =* .32, *p* < .001) and increased psychological distress (*r =* .46, *p* < .001) (Table 4). Similarly, problematic gaming was also associated with decreased sleep quality (*r =* .22, *p* < .001) and increased psychological distress (*r =* .46, *p* < .001) (Table 4).

**Table 4:** Bivariate correlation between problematic internet use (PIU), problematic gaming, sleep quality, and psychological distress (*N =* 1040)

	1	2	3	4
1.Problematic internet use	1.00			
2.Problematic gaming	0.65^***^	1.00		
3.Sleep quality	0.32^***^	0.22^***^	1.00	
4.Psychological distress	0.46^***^	0.46^***^	0.47^***^	1.00

^***^p < 0.001

### Mediation analysis

Mediation analysis was first conducted with PIU as the exposure variable (Table 5). In the unadjusted model, 25.7% of the indirect effect of PIU on psychological distress was mediated by sleep quality. After adjusting for sociodemographic confounders in Model 2, the proportion of indirect effect via sleep quality decreased to 24.9%. When behavioral factors (physical activity and substance use) were further accounted for in model 3, the proportion of indirect effect via sleep quality was reduced to 23.5%. In all three models, sleep quality exerted partial but statistically significant effects on the PIU-psychological distress link.

**Table 5:** Results from mediation analysis

	Model 1		Model 2		Model 3	
	B (SE)	BC 95% CI	B (SE)	BC 95% CI	B (SE)	BC 95% CI
Sleep quality mediates the association between problematic internet use (PIU) and psychological distress (*N* = 1040)						
Indirect effect	0.47 (0.06)	0.36, 0.60	0.45 (0.06)	0.34, 0.57	0.36 (0.05)	0.26, 0.47
Direct effect	1.37 (0.11)	1.16, 1.59	1.36 (0.11)	1.15, 1.57	1.17 (0.10)	0.97, 1.37
Total effect	1.85 (0.11)	1.63, 2.07	1.81 (0.11)	1.59, 2.03	1.53 (0.11)	1.32, 1.73
Proportion of total effect mediated^a^	25.66%		24.87%		23.50%	
Ratio of indirect to direct effect^a^	34.52%		33.11%		30.72%	
Sleep quality mediates the association between problematic gaming and psychological distress (*N* = 1040)						
Indirect effect	0.81 (0.13)	0.56, 1.07	0.81 (0.13)	0.56, 1.08	0.59 (0.12)	0.36, 0.85
Direct effect	3.51 (0.24)	3.05, 3.98	3.57 (0.25)	3.09, 4.06	2.72 (0.25)	2.24, 3.20
Total effect	4.32 (0.26)	3.82, 4.83	4.38 (0.27)	3.86, 4.90	3.31 (0.27)	2.79, 3.83
Proportion of total effect mediated^a^	18.67%		18.45%		17.89%	
Ratio of indirect to direct effect^a^	22.96%		22.62%		21.78%	

^a^All percentages were derived from 4-digit values and rounded to two decimal places

Model 1: unadjusted mediation model

Model 2: adjusted for sociodemographic (gender, class standing, academic achievement, annual household income, father’s education, mother’s education)

Model 3: adjusted for sociodemographic + behavioral variables (gender, class standing, academic achievement, annual household income, father’s education, mother’s education + level of physical activity and substance use)

*BC 95% CI* Bias-corrected confidence intervals based on 10,000 bootstrap samples, *B* unstandardized coefficient, *SE* standard error

Mediation analysis was also conducted with problematic gaming as the exposure variable (Table 5). In the unadjusted model, 18.7% of the indirect effect of problematic gaming on psychological distress was mediated via sleep quality. After adjusting for sociodemographic confounders in model 2, the proportion of indirect effect via sleep quality decreased to 18.5%. And after behavioral factors were further adjusted for in model 3, the proportion of indirect effect via sleep quality was reduced to 17.9%. In all three models, sleep quality exerted partial but significant mediating effects on problematic gaming-psychological distress link.

## Discussion

This study was the first to examine the mediating role of sleep quality in the PIU- and problematic gaming-psychological distress link among college students in China. Findings revealed that both PIU and problematic gaming was associated with decreased sleep quality and increased psychological distress. The main finding was that, sleep quality explained 23.5% of the total effect of PIU on psychological distress, and only 17.9% of the total effect of problematic gaming on psychological distress. Thus, sleep quality was deemed a meaningful mediator only in the PIU-psychological distress link. This finding implied that, PIU may increase psychological distress directly or indirectly through decreasing sleep quality; whereas problematic gaming had a strong direct negative influence on psychological distress, and the influence can barely be adequately explained via decreased sleep quality.

This finding may be better understood through the following mechanisms. First, existing evidence seems PIU-sleep relation has been well-documented, whereas the problematic gaming-sleep relation seemed to be less consistent. Excessive internet use has been associated with a wide range of sleep problems, such as reduced sleep duration, sleep disorders, insomnia, frequent sleep disturbances, increased fatigue and drowsiness during the day [54]. A systematic review found that, compared with those without PIU, those with PIU had 2.2 times increased odds (pooled) of having sleep problems, and a 15-min reduction in sleep duration [54]. In contrast, one systematic review examined both the PIU-sleep and problematic gaming-sleep relations, finding strong evidence for the PIU-sleep relation, but insufficient evidence supporting the problematic gaming-sleep relation [34]. In another systematic review, 18 out of 21 studies found a significant association between video gaming and sleep outcomes, and only 1 out of 3 studies specifically focusing on sleep quality found a significant association between problematic gaming and sleep quality [55]. Second, factors other than sleep quality might contribute significantly to the problematic gaming-psychological distress relation. For example, problematic gaming may strongly reflect individual characteristics such as high neuroticism and external locus of control, which were also highly associated with psychological distress but not via pathways related to sleep [56].

Griffiths has argued that a distinction should be made between addictions on the internet and addictions to the internet, as internet can be used as a medium or vehicle to fuel other obsessive behaviors such as gambling, gaming, and pornography [7]. More specifically, a distinction should be made about “generalized internet addiction” and “specific internet addiction” [7]. Following this logic, problematic gaming behavior maybe best conceptualized as a behavior distinct from PIU. This proposition has been empirically tested in a 2014 study using a nationally representative sample of adolescent gamers in Hungary, the authors found PIU was positively associated with online gaming, online chatting, and social networking, while problematic gaming was only associated with being male and online gaming [57], lending support to the notion that PIU and problematic gaming were distinct entities. In a second study, the authors examined generalized and specific internet use from college students in Germany, Sweden, China, and Taiwan, and found that except for problematic social network use, the correlation between generalized PIU and all other specific types of PIU were substantially lower or non-significant [58], implying the uniqueness of specific types of PIU. The main finding of the present study that PIU and problematic gaming were differentially related to psychological distress via sleep quality may have lent further support for the conceptual distinction between PIU and problematic gaming.

Findings on sociodemographic covariates in this study may also have implications for PIU and problematic gaming. Consistent with existing research [59], the mean score of problematic gaming was found to be significantly higher among male students, indicating that more males engaged in problematic gaming than females. This gender difference might be explained by craving-related neural mechanisms underlying problematic gaming, such that the same gaming stimulation may elicit more craving-related activation in males rather than females [59]. Internet use and problematic gaming have generally been associated with academic under-achievement, yet, some studies suggested that they had little impact on non-pathological users [60]. In this study, the mean score of PIU and problematic gaming were both found to be highest among those with poor academic achievement (bottom 20% of the class), yet, due to the cross-sectional nature of the study, causal inferences cannot be drawn. Two indicators of respondents’ socioeconomic status were examined in this study: annual family income (direct indicator) and parental education level (proxy indicator). Annual family income was found to be associated with PIU but not with problematic gaming, and the mean score of PIU was highest among students reporting the lowest family income. While both parents’ highest level of education was associated with PIU as well as problematic gaming, and the mean scores of both PIU and problematic gaming were lowest among respondents with college-educated parents. This indicated that parental education level might be a better indicator of PIU and problematic gaming. However, parental education level has been rarely included in studies on PIU and problematic gaming, and findings from only a few studies across different populations offered inconclusive evidence [61, 62]. In this study, the mean scores of both PIU and problematic gaming were also found to be higher among those that used any type of substance, and highest among those who used at least 2 types of substances in the past year. Problematic internet use, problematic gaming behaviors, and substance use disorder are considered to share many core features and comorbidities, there is also evidence supporting substantial similarities in the neurobiological mechanisms underlying each type of disorder [63]. Findings of this study confirmed their strong associations among college students in China, suggesting that strategies to prevent or reduce PIU or problematic gaming may include those targeting substance use prevention. However, it remains to be determined whether prevention or treatment efforts intended for both conditions are more effective.

### Limitations

Nonetheless, this study had several limitations. First, mediation analysis was performed using cross-sectional data, thus, causal inferences cannot be drawn. However, it is worth to note that the respective etiology of psychological distress, PIU, problematic gaming, and poor sleep quality is likely multifactorial, with complex interplay between a multitude of intrinsic and external factors. Examining these factors or understanding how they would interact overtime was beyond the scope of the current study. Our mediation model echoed the developing consensus in the literature that problematic gaming may perhaps be a distinct concept from PIU, as reflected in the growing international recognition of IGD rather than PIU as a mental disorder. Nonetheless, results of our study warrant the need for further longitudinal studies to substantiate the temporal sequence of these associations. Second, in the present study, problematic gaming was assessed by the GAS-7, which was developed precedes the DSM-V, and may suffer from incomplete coverage of diagnostic criteria of IGD outlined in ICD-11 or DSM-V [64]. However, the GAS-7 is considered a screening tool, and was found to be the most common tool used in prevalence as well as longitudinal studies to assess problematic gaming, and exhibited strong psychometric properties [64]. Further studies utilizing instruments consistent with ICD-11 classification are needed nonetheless. Third, sleep quality was measured using self-report PSQI, which might be subject to recall bias or social desirability bias. Future studies may benefit from comparing findings using both subjective and objective measures of sleep quality.

## Conclusion

The study revealed that sleep quality had a meaningful mediation effect on the PIU-psychological distress link, but exerted less consequential mediating effect on the problematic gaming-psychological distress link. The findings implied that the association of PIU with increased psychological distress can be partly attributed to decreased sleep quality in a meaningful way. In addition to promoting healthy internet usage, strategies aimed at mitigating the negative effect of excessive internet use on psychological health might benefit from those aimed at improving sleep quality (i.e. establishing a consistent bedtime routine, applying stimulus control before bedtime, and associating the bedroom with sleep [65]. Although results on sociodemographic covariates in relation to PIU and problematic gaming indicated that academic achievement and parental education level were both significant correlates, further research is needed to examine if their associations would vary in same-age populations across different countries, as well as to explore the underlying mechanisms contributing to the variations.

## Data Availability

The datasets used and/or analyzed during the current study are available from the corresponding author on reasonable request.
